# Cysteine-mediated decyanation of vitamin B12 by the predicted membrane transporter BtuM

**DOI:** 10.1038/s41467-018-05441-9

**Published:** 2018-08-02

**Authors:** S. Rempel, E. Colucci, J. W. de Gier, A. Guskov, D. J. Slotboom

**Affiliations:** 1 Groningen Biomolecular and Biotechnology Institute (GBB), University of Groningen, Nijenborgh 4, 9474 AG Groningen, The Netherlands; 2 Department of Biochemistry and Biophysics, Stockholm University, 10691 Stockholm, Sweden; 3 Zernike Institute for Advanced Materials, University of Groningen, Nijenborgh 4, 9747 AG Groningen, The Netherlands

## Abstract

Uptake of vitamin B12 is essential for many prokaryotes, but in most cases the membrane proteins involved are yet to be identified. We present the biochemical characterization and high-resolution crystal structure of BtuM, a predicted bacterial vitamin B12 uptake system. BtuM binds vitamin B12 in its base-off conformation, with a cysteine residue as axial ligand of the corrin cobalt ion. Spectroscopic analysis indicates that the unusual thiolate coordination allows for decyanation of vitamin B12. Chemical modification of the substrate is a property other characterized vitamin B12-transport proteins do not exhibit.

## Abstract (web summary)

Uptake of vitamin B12 is essential for many prokaryotes, but in most cases the membrane proteins involved are yet to be identified. Here, the authors use X-ray crystallography and spectroscopy to characterize BtuM, a predicted bacterial substrate-modifying vitamin B12 transporter.

## Introduction

**Fig. 1:**
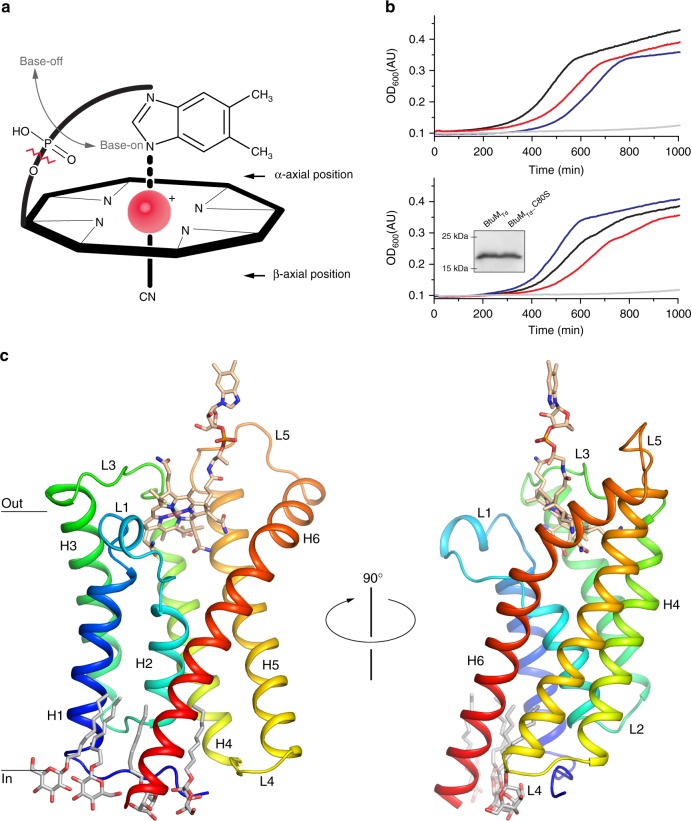
Function and structure of BtuM_Td_. **a** Schematic representation of cobalamin (Cbl) showing the corrinoid ring with the central cobalt ion (red). The ligand at the β-axial position is in this case a cyano-group, but differs in various Cbl variants (Supplementary Figure 1a, b). The ligand at the α-axial position (base-on conformation) is the 5,6-dimethylbenzimidazole base, which is covalently linked to the corrinoid ring. When this coordination is lost, Cbl is termed base-off. Cbi lacks the 5,6-dimethylbenzimidazole base (indicated by the zigzagged red line). **b** Growth assays with *E. coli* ΔFEC was conducted in the presence of 50 μg ml^−1^ l-methionine or 1 nM Cbl. Additional experiments in the presence of different Cbl concentration are shown in Supplementary Figure 2f and g. All growth curves are averages of nine experiments (three biological triplicates, each with three technical replicates). Top panel: cells containing the empty expression vector (pBAD24) in the presence of methionine (blue line) or Cbl (grey line) and cells expressing the BtuCDF system (black and red lines, respectively). Bottom panel: cells expressing BtuM_Td_ (black and red lines) or mutant BtuM_Td__C80S (blue and grey) in the presence of methionine and Cbl, respectively. The inset displays a western blot showing that the mutant is expressed to wild-type levels (the full-length western blot can be found in Supplementary Figure 2h). **c** The structure of BtuM_Td_ in cartoon representation, coloured from blue (N terminus) to red (C terminus) and viewed from the membrane plane. α-helices (H1-6) and connecting loops (L1-5) are indicated. Cbl is shown in stick representation with carbon atoms coloured wheat, the oxygen and nitrogen atoms in red and blue, respectively, the cobalt ion in pink. Four *n*-nonyl-β-d-glucopyranoside detergent molecules are also shown in stick representation (carbons in light grey)

Cobalamin (Cbl) is one of the most complex cofactors (Supplementary Figure 1a) known, and used by enzymes catalyzing for instance methyl-group transfer and ribonucleotide reduction reactions^1,2^. For example, in the methionine synthase MetH, the cofactor is used to transfer a methyl moiety onto l-homocysteine to produce l-methionine^1,3^. Many bacteria require Cbl for survival^1,2,4,5^, but only a small subset of prokaryotic species can produce this molecule de novo, via either an aerobic or anaerobic pathway^4,5^. Roughly two thirds of archaea and eubacteria are Cbl-auxotrophs that rely on uptake of either Cbl or its precursor cobinamide^2,5,6^ (Cbi, Fig. 1a, Supplementary Figure 1b). Dependence on uptake has probably evolved, because synthesis of Cbl involves roughly 30 different enzymes and is energetically costly. Gram-negative bacteria require the TonB-dependent transporter BtuB^1^ to transport Cbl across the outer membrane (Supplementary Figure 1c). For subsequent transport of vitamin B12 across the cytoplasmic membrane, the only characterized bacterial uptake system is the ABC transporter BtuCDF, which is predicted to be present in approximately 50% of Cbl-auxotrophic bacteria^5,7^. Many Cbl-auxotrophic Gram-negative bacteria do not encode BtuCDF, whereas they do contain BtuB. Metabolic reconstruction and chromosomal context analyses, e.g. co-localization with the gene for BtuB, have identified potential alternative inner membrane vitamin B12 transporters, one of which is BtuM^5^. BtuM homologues are small membrane proteins of ~22 kDa, and found predominantly in Gram-negative species, distributed mostly over α-, β-, and γ-proteobacteria (Supplementary Data 1).

Here, we sought to characterize the predicted vitamin B12 transporter BtuM from *Thiobacillus denitrificans* (BtuM_Td_). We show that BtuM_Td_ is involved in transport of Cbl in vivo and we solved its structure to 2 Å resolution. A cobalt–cysteine interaction allows for chemical modification of the substrate prior to translocation, which is a rare feature among uptake systems.

## Results

### BtuM_Td_ supports vitamin B12-dependent growth

To test experimentally whether BtuM_Td_ is a potential Cbl-transporter, we constructed an *Escherichia coli* triple knockout strain, *E. coli* ΔFEC, based on Cadieux et al.^8^. In this strain, the gene encoding the Cbl-independent methionine synthase, MetE^9^, is deleted. The *metE* deletion makes it impossible for *E. coli* ΔFEC to synthesize methionine, unless it can import Cbl^8,9^. In that case, l-methionine can be synthesized using the Cbl-dependent methionine synthase, MetH^3,8^. *E. coli* ΔFEC has additional deletions in *btuF* and *btuC*, encoding subunits of the endogenous Cbl-transporter BtuCDF^7,8^. Therefore, *E. coli* ΔFEC cannot import Cbl, prohibiting MetH-mediated l-methionine synthesis. Consequently, *E. coli* ΔFEC can grow only if l-methionine is present or if vitamin B12 import is restored by (heterologous) expression of a Cbl-transport system^8^. The phenotype of *E. coli* ΔFEC was confirmed in growth assays (Supplementary Figure 2a). Cells that are not expressing any Cbl-transporter did not exhibit substantial growth in methionine-free medium, whereas cells complemented with an expression plasmid for BtuCDF grew readily (Fig. 1b). Cells expressing BtuM_Td_ had a similar growth phenotype, indicating that BtuM_Td_ is a potential transporter for vitamin B12 (Fig. 1b).

### Crystal structure of BtuM_Td_ bound to vitamin B12

The BtuM family contains an invariably conserved cysteine residue (Supplementary Figure 3a). In BtuM_Td_, this cysteine is located at position 80, and mutation to serine abolishes the ability of the protein to complement the *E. coli* ΔFEC strain (Fig. 1b). To investigate the role of the cysteine, we solved a crystal structure at 2 Å resolution of BtuM_Td_ in complex with Cbl. Data collection as well as refinement statistics are summarized in Table 1. BtuM_Td_ consists of six transmembrane helices with both termini located on the predicted cytosolic side (Fig. 1c). The amino acid sequences of BtuM proteins are not related to any other protein^5^ but, surprisingly, BtuM_Td_ resembles the structure of S-components from energy-coupling factor (ECF)-type ABC transporters^10^ (Supplementary Figure 4 and Supplementary Table 1). In contrast to BtuM proteins, ECF-type ABC transporters are predominantly found in Gram-positive bacteria. They are multi-subunit complexes consisting of two peripheral ATPases and two transmembrane components (EcfT and S-component)^10,11^. EcfT and the ATPases together form the so-called ECF-module. S-components bind the transported substrate, and dynamically associate with the ECF-module to allow substrate translocation^10,12–14^. Intriguingly, no homologues of EcfT could be found in *T. denitrificans*. In addition, all ABC-type ATPases encoded by the organism are predicted to be part of classical ABC transporters, and not ECF transporters. Therefore, we conclude that the organism does not encode an ECF-module, and hypothesize that solitary BtuM_Td_ may be responsible for Cbl uptake. This hypothesis is supported by the ability of BtuM_Td_ to transport vitamin B12 when expressed heterologously in *E. coli* ΔFEC. Importantly, *E.coli* also does not encode an ECF-module^11^, hence BtuM_Td_ cannot interact with a module from the host, and BtuM_Td_ must be able to support Cbl uptake using a different mechanism than that of ECF transporters^10,11^. In a few cases, the biotin-specific S-component BioY^15^ has also been found in organisms lacking an ECF-module and was shown to mediate transport without the need for an ECF-module^15^. However, organisms encoding only BioY without an ECF-module are rare^15^, and in the large majority of organisms BioY is associated with an ECF-module^11^. In contrast, BtuM homologues (apart from one exception) are found exclusively in organisms lacking an ECF-module (Supplementary Data 1).

**Table 1:** Data collection and phasing and refinement statistics

	Cbl-bound BtuM_Td_ native	Cbl-bound BtuM_Td_ anomalous
Data collection		
# Crystals/# datasets	1/1	1/2
Space group	P 31 2 1	P 31 2 1
Unit cell dimensions		
*a, b, c* (Å)	87.54, 87.54, 97.91	86.60, 86.60, 97.51
*α, β, γ* (°)	90.0, 90.0, 120.0	90.0, 90.0, 120.0
Resolution range (Å)	41.13–2.01 (2.082–2.01)^a^	43.30–2.50 (2.5896–2.5002)^a^
*R*_merge_ (%)	5.8 (>100)^a^	10.8 (>100)^a^
*cc*_1/2_	100.0 (14.1)^a^	99.9 (50.8)^a^
*I*/*σI*	16.24 (0.23)^a^	18.23 (1.53)^a^
Completeness (%)	99.9 (99.8)^a^	93.82 (64.7)^a^
Redundancy	10.5 (9.7)^a^	18.8 (11.0)^a^
Refinement		
Resolution (Å)	41.13–2.01	43.30–2.50
No. of reflections	28,953	14,144
*R*_work_/*R*_free_	0.2121/0.2338	0.2492/0.2854
Number of non-hydrogen atoms	1870	1536
Protein	1640	1359
Ligands	208	177
Water	22	0
*B*-factors		
Protein	89.0	67.2
Cobalamin	65.4	69.3
PEG	106.2	—
Detergent	107.1	—
Water	69.2	—
R.m.s. deviations		
Bond lengths (Å)	0.009	0.009
Bond angles (°)	1.81	1.916

^a^Values in parentheses are for the highest-resolution shell

Further experiments, for instance using purified protein reconstituted in proteoliposomes, are required to test whether BtuM_Td_ also catalyses transport in vitro without any additional component involved. However, the in vivo assay gives a very strong indication that BtuM_Td_ is a transporter itself, as the protein was expressed in a heterologous host that does not contain any ECF-module or S-component. Similar in vivo experiments have been used extensively in the past to identify other transporters (for instance ref.^16^) and have the advantage over in vitro assays that physiologically relevant conditions are used.

### BtuM_Td_ binds cobalamin using cysteine ligation

**Fig. 2:**
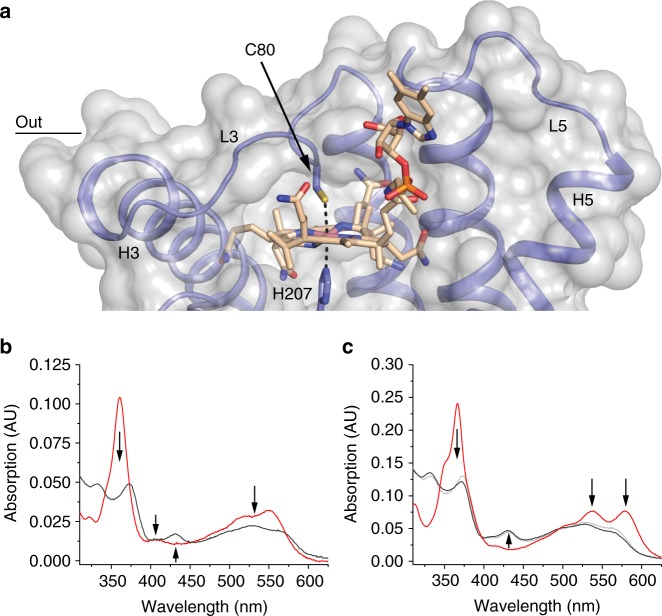
Binding of vitamin B12 by BtuM_Td_. **a** Transparent surface representation (light grey) of the binding pocket of BtuM_Td_ with bound Cbl. The protein backbone is shown in blue. The Co-ion is coordinated by Cys80 located in L3 (Co to sulphur distance 2.7 Å) and His207 (Co to nitrogen distance 2.4 Å) from a neighbouring symmetry mate (Supplementary Figure 6). A complete description of the interactions of BtuM_Td_ with its substrate can be found in Supplementary Figure 12. **b** The spectrum of BtuM_Td__cHis8-bound Cbl (4.3 μM, black line) compared to unbound cyano-Cbl (2.4 μM, red line). The regions of the spectrum with major changes are indicated with arrows. **c** Same as **b** but with Cbi bound to the protein (9.2 μM black line), compared to unbound dicyano-Cbi (9 μM, red line). The regions of the spectrum with major changes are indicated with arrows. For comparison, a scaled spectrum of Cbl bound to BtuM_Td_ (light grey line) from **b** is included, showing that the spectrum of both substrates bound to the protein is virtually the same, indicating the same binding mode

Close to the predicted periplasmic surface of BtuM_Td_, we found well-defined electron density (Supplementary Figure 5) representing a bound Cbl molecule. The binding mode of Cbl in the crystal structure (Fig. 2a) is striking for two reasons. First, the essential Cys80 is the α-axial ligand of the cobalt ion. To our knowledge, cobalt coordination by cysteine has not been observed in any other Cbl-binding protein of known structure. Binding of cysteine to cobalt in a corrinoid has been hypothesized for the mercury methylating enzyme HgcA^17^ and observed in a synthetic cyclo-decapeptide, but in the latter case the residue replaced the β-ligand^18^.

Second, Cbl is bound to BtuM_Td_ in the base-off conformation in which the 5,6-dimethylbenzimidazole moiety does not bind to the cobalt ion (Fig. 2a). In contrast, at physiological pH the conformation of free Cbl in aqueous solution is base-on with the 5,6-dimethylbenzimidazole moiety coordinated to the cobalt ion in the α-axial position^1^ (Fig. 1a). The base-off conformation has been found only in a subset of Cbl-containing enzymes, but not in Cbl-binding proteins without enzymatic activity^1^, such as the periplasmic substrate-binding protein BtuF^19^, the outer membrane transporter BtuB^20^, and human Cbl-carriers intrinsic factor^21^, haptocorrin^22^ and transcobalamin^23^. Enzymes that bind Cbl with the base-off conformation usually use a histidine residue as the α-axial ligand. In this way, the reactivity of the cobalt at the β-axial position is altered, allowing among others a variety of methyl-group transfer reactions^1^. Therefore, the base-off binding mode by BtuM_Td_ could indicate that the protein may exhibit enzymatic activity.

### BtuM_Td_ catalyses decyanation of vitamin B12

Indeed, the structure of BtuM_Td_ suggests that the protein can catalyse chemical modification of the substrate. We co-crystallized BtuM_Td_ with cyano-Cbl, which contains a cyano-group as the β-ligand^1,4^. Cyano-Cbl is the most stable form of vitamin B12^4^ but, despite the tight binding of the β-ligand, in the crystal structure the cyano-group is absent indicating protein-mediated decyanation. Consistent with decyanation and the presence of a cysteine ligand in BtuM_Td_, the absorbance spectrum of Cbl-bound BtuM_Td_ showed pronounced differences compared to that of free Cbl^18,24^ (Fig. 2b). The characteristic absorption peak at 361 nm of Cbl is absent and two peaks with lower absorption appear around 330 and 370 nm. The absorption between 500 and 580 nm is lower than in free Cbl, and a new peak at 430 nm is present.

**Fig. 3:**
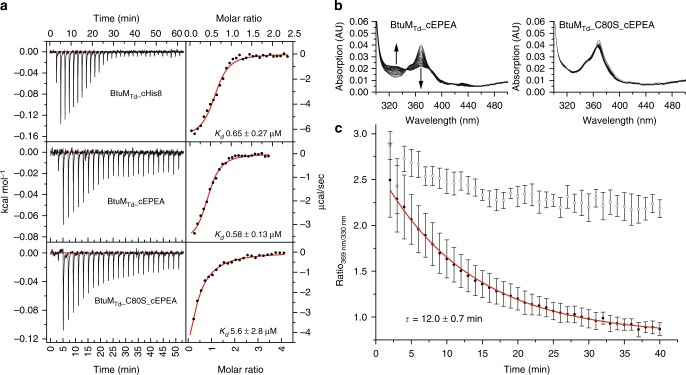
Cobinamide (Cbi) binding to BtuM_Td_ and BtuM_Td_-catalysed decyanation. **a** Representative ITC-measurements of differently tagged BtuM_Td_ constructs. BtuM_Td_ with a C-terminal His-tag binds Cbi with a *K*_d_ value of 0.65 ± 0.27 μM (top). EPEA-tagged BtuM_Td_ binds Cbi with essentially the same affinity of *K*_d_ 0.58 ± 0.13 μM (middle). For the EPEA-tagged mutant version BtuM_Td__C80S *K*_d_ = 5.6 ± 2.8 μM (bottom). All ITC experiments were performed as technical triplicates, error is s.d. **b** Decyanation of Cbi catalysed by EPEA-tagged BtuM_Td_. Upon addition of an excess of BtuM_Td_ to Cbi, the substrate is slowly decyanated, which can be followed spectroscopically (left) with the main spectral changes indicated by the arrows. The mutant BtuM_Td__C80S, did not catalyse decyanation (right). **c** Quantification (error bars are s.d. of technical triplicates) of decyanation reveals that the process is slow. The ratio of the absorption at 369 nm over 330 nm of BtuM_Td_ (black dots) was plotted as function of time. A mono-exponential decay function was fitted to the data (red line) to extract *τ* = 12 ± 0.7 min (s.d. of technical triplicates), which is comparable to the decyanation rate of the His-tagged protein and the process follows pseudo-first order kinetics (Supplementary Figure 8b, c). The ratio of absorption obtained with the cysteine mutant (open dots), which does not catalyse decyanation, is shown for comparison

In place of the cyano-group, the imidazole group of His207 from a neighbouring BtuM_Td_ molecule in the crystal is located at the β-axial position. His207 is the last histidine residue of the His_8_ affinity-tag (His-tag) engineered at the C terminus of the protein (Supplementary Figure 6). Because crystal contacts may be non-physiological and the His-tag is a non-natural addition to the protein, we performed control experiments to exclude the possibility that decyanation is an artefact. First, we showed by mass spectrometry (MS) that the loss of the cyanide does not require crystal formation (Supplementary Figure 7a). Second, we showed that decyanation also occurred by BtuM_Td_ with a C-terminal Glu-Pro-Glu-Ala (EPEA)-tag instead of a His-tag (Supplementary Figure 7b). Notably, the EPEA-tagged protein was active in the growth assay and also removal of the His-tag did not affect activity (Supplementary Figure 2b, c). Finally, binding of Cbl to BtuM_Td_ with His-tag or EPEA-tag was accompanied by the same changes in absorption spectrum (Figs. 2b, 3b). Therefore, we conclude that decyanation takes place regardless of crystal formation or presence of a His-tag.

### Kinetics of the BtuM_Td_ catalysed decyanation reaction

To study the kinetics of BtuM_Td_-catalysed decyanation we used cobinamide (Cbi) instead of Cbl as substrate. Because Cbi does not contain the 5,6-dimethylbenzimidazole moiety (Fig. 1a), it mimics the base-off conformation of cobalamin, which makes the compound suitable to study decyanation without interference from the slow conversion^25^ of base-on to base-off Cbl. The absorption spectra of Cbl-bound and Cbi-bound BtuM_Td_ are almost identical (Fig. 2c), indicating identical coordination of the cobalt ion of Cbi at the α-axial and β-axial positions. MS analysis showed that binding of Cbi to BtuM_Td_ also results in decyanation (Supplementary Figure 8a). To probe Cbi binding by BtuM_Td_, we used isothermal titration calorimetry (ITC), which revealed dissociation constants for the His-tagged and EPEA-tagged protein of 0.65 ± 0.27 and 0.58 ± 0.13 μM (s.d. of the mean of technical triplicates), respectively (Fig. 3a). It is noteworthy that we were unable to assay for Cbl-binding by ITC. We speculate that the conversion from base-on to base-off Cbl is so slow^25^ that it may prevent detection of Cbl-binding by ITC. Additionally, the absence of the membrane environment also appears to preclude Cbl binding to purified BtuM_Td_, as binding was observed only when the substrate was added before solubilisation (Fig. 2b, c, Supplementary Figure 9).

Because binding of cyanide to cobinamide causes a decrease in absorbance at 330 nm and an increase at 369 nm^24^, we expected the opposite spectral changes upon decyanation. Addition of excess of *apo*-BtuM_Td_ (Supplementary Figure 10a and b) to a solution of Cbi indeed revealed time-dependent changes in absorbance consistent with a decyanation reaction (Fig. 3b, c). Decyanation occurred with an apparent time constant of τ = 12 ± 0.7 min (s.d. from technical triplicates, Fig. 3c), which is comparable to the rate observed in the human decyanating enzyme CblC^25,26^. We also tested Cbi binding and decyanation using mutant proteins C80A and C80S. While these mutants were unable to bind Cbl, they remained capable of binding Cbi as demonstrated by co-purification of the molecule with the protein (Supplementary Figure 10c, d). We measured the affinity of BtuM_Td__C80S to Cbi with ITC and found a dissociation constant of 5.6 ± 2.8 μM (s.d. of the mean of technical triplicates), which is an order of magnitude weaker than the wild-type (WT) protein. The absorbance spectra of Cbi bound to the mutant proteins showed the characteristic features for cyano-Cbi, indicating that decyanation was abolished (Supplementary Figure 10c, d). Consistently, the decyanation assay with BtuM_Td__C80S did not reveal the slow spectral changes observed for the WT protein (Fig. 3b, c). These results show that Cys80 is required for decyanation of Cbi and that binding and modification of the substrate are separate events: fast binding (detected by ITC) is followed by slow modification. The lack of detectable binding of Cbl to BtuM_Td__C80S (measured by lack of co-purification, Supplementary Figure 10c, d) may indicate that the cysteine is also required for base-on to base-off conversion, and that the base-on conformer binds with too low affinity for detection by co-purification. To understand BtuM_Td_-catalysed decyanation of Cbl and Cbi in more detail, we mutated conserved amino acids H28, D67, Y85, and R153 located in the binding pocket (Supplementary Figure 3b). Mutant D67A could not be purified, and was not analysed further. Cbl-bound mutants H28A, Y85L, and R153A displayed the same spectral properties as the WT protein (Supplementary Figure 11a), and MS analysis showed that the binding of Cbl was accompanied by decyanation, indicating that the conserved residues are not essential for the reaction (Supplementary Figure 11b–d). Finally, to exclude that BtuM_Td_ is merely a decyanating enzyme, and that the potential reaction product hydroxyl-Cbl is subsequently transported by another protein, we show that BtuM_Td_ also mediates uptake of hydroxyl-Cbl in the growth assay (Supplementary Figure 2d, e).

## Discussion

We showed in vivo that BtuM_Td_ is a vitamin B12 transporter, which is consistent with the predictions based on bioinformatics analysis^5^. Our work sheds light on the diversity of transport systems used for the uptake of vitamin B12. The outer membrane transporter BtuB is a TonB-dependent active transporter, which uses a different mechanism of transport than inner membrane proteins^1,20^. The well-studied inner membrane type II ABC transporter BtuCDF uses hydrolysis of ATP to pump Cbl into the cell like the ECF-transporter, ECF-CbrT^27^. Both systems require a substrate-binding protein and are multiprotein complexes^7,8,27,28^. BtuM_Td_ on the other hand, must operate by a different mechanism because the protein lacks accessory components and the expected ATPase motifs of ABC transporters^10^. BtuM_Td_ structurally resembles the S-components of ECF transporters. In ECF transporters, the S-components bind the transported substrate with high affinity and then associate with an ECF-module for energizing transport. During the transport cycle, the S-components rotate (topple over) in the membrane to bring the substrate from the outside to the cytoplasm. We hypothesize that BtuM_Td_ mediates the translocation of Cbl through the membrane by a similar toppling mechanism. Because BtuM_Td_ does not require an ECF-module, the transport mode may be facilitated diffusion along the concentration gradient of the substrate. In *T. denitrificans* and most other BtuM hosts, the BtuM_Td_ gene co-localizes with *btuR*, which encodes for the cobalamin adenosyltransferase BtuR. This enzyme catalyses the synthesis of 5′-deoxyadenosyl-cobalamin and would offer a mechanism of metabolic trapping, similar to what has been proposed for other vitamin transporters in bacteria^29^.

Our work provides experimental evidence for a binding mode of Cbl, in which cysteine ligation and the base-off conformation are linked. This binding mode leads to decyanation of cyano-Cbl, for which we propose a reductive decyanation mechanism, which depends on Cys80^17,18^ (Supplementary Figure 8d). The proposed decyanation mechanism differs from the mechanism used by CblC, where a flavin acts as reducing agent. In CblC, the flavin donates two electrons resulting in the reductive decyanation (CN^−^) and the reduction of the Co-ion^25,26^. For BtuM_Td_, cysteine-catalysed reductive decyanation would only result in the release of CN^−^, but not in the reduction of the Co-ion.

Finally, BtuM_Td_ likely combines two functions: transport of the substrate into the bacterial cell, and chemical modification of the substrate. Such combined functionality rarely occurs in transporters, and has been observed only in phosphotransferase systems^30^. However, in that case the modification (phosphorylation) takes place on the cytoplasmic side of the membrane^30^, whereas BtuM_Td_ appears to modify on the periplasmic side of the membrane. Internalisation of decyanated vitamin B12 may be relevant because environmental cyano-Cbl exists^26^. A combination of decyanation and transport activity would make cyano-Cbl directly accessible for conversion into physiological forms, for example, by BtuR.

## Methods

### Bioinformatic identification of BtuM homologues and ECF-modules

The amino acid sequence of BtuM_Td_ was used as a search query using the iterative jackHMMer algorithm (default settings) with the reference proteome database^31^ until the search converged leading to 131 hits. Within the genomes of the identified 131 organisms, we screened for the presence of an ECF-module using the pHMMer algorithm (default settings)^31^ with the amino acid sequence of the transmembrane component (ECF-T) from *Lactobacillus delbrueckii*^14^ as a search query. Additionally, we used the SEED viewer (http://pseed.theseed.org) to verify the absence of any ECF-transporter in a subset of organisms (46 present in the SEED database) and also used this tool to find all ABC transporters in *T. denitrificans* to verify that none of these are an ECF-transporter.

### Molecular methods

For expression in *E. coli* MC1061^32^ a codon optimized version (Invitrogen) of *btuM* (Tbd_2719) from *Thiobacillus denitrificans* ATCC25259 with a C-terminal eight histidine affinity-tag or EPEA-tag was used and introduced into pBAD24^33^ with *Nco*I and *Hin*dIII restriction sites. A single glycine (Gly2) was introduced to be in-frame with the start-codon of the *Nco*I restriction site. Single amino acid substitutions and removal of the affinity tag were conducted using site-directed mutagenesis. The complementation plasmid for expression of BtuC and BtuF was constructed using Gibson Assembly following the standard procedure (NEB). All constructs were checked for correct sequences by DNA sequencing. All primers are listed in Supplementary Table 2.

### Construction of the ΔFEC strain

*E. coli* ΔFEC, was constructed by P1-mediated generalized transduction^27,34,35^. In short: *E. coli* JW0154 (*ΔbtuF::Km*^*R*^) was used as the basis for construction of *E. coli* ΔFEC. The kanamycin resistance cassette was removed using the FLP-recombinase^36^. The *metE::Km*^*R*^ locus from *E. coli* JW3805 and the *ΔbtuC::Km*^*R*^ locus of *E. coli* JW1701 was introduced^34,35^, resulting in *E. coli* ΔFEC (*ΔbtuF, ΔmetE, ΔbtuC::Km*^*R*^*)*. Colony PCRs based on three primer pairs^27^ were used to verify *Km*^*R*^-insertions, FLP-recombinase-mediated removal of *Km*^*R*^-markers and absence of genomic duplications (Supplementary Figure 13).

### Growth assays

The strains carrying various expression vectors were grown overnight at 37 °C on LB-agar plates supplemented with 25 μg ml^−1^ kanamycin and 100 μg ml^−1^ ampicillin. M9 minimal medium (47.7 mM Na_2_HPO_4_ × 12H_2_O, 17.2 mM KH_2_PO_4_, 18.7 mM NH_4_Cl, 8.6 mM NaCl) was supplemented with 0.4% glycerol, 2 mM MgSO_4_, 0.1 mM CaCl_2_, 100 μg ml^−1^ l-arginine, 25 μg ml^−1^ kanamycin and 100 μg ml^−1^ ampicillin. A single colony was picked and used to inoculate an M9-medium pre-culture supplemented with 50 μg ml^−1^ l-methionine (Sigma-Aldrich). The pre-culture was grown ~24 h at 37 °C, shaking in tubes with gas-permeable lids (Cellstar), and then used to inoculate the assay medium in a 1:500 ratio. The assay medium was supplemented with 0.00001% l-arabinose (Sigma-Aldrich) and either 50 μg ml^−1^ l-methionine, 0.01 nM, 1 nM and 5 nM cyano-cobalamin (Acros Organics), or 0.1 nM hydroxy-cobalamin (Sigma-Aldrich). Overall, 200 μl medium was added per well of a sterile 96 well plate (Cellstar). Plates were sealed with a sterile and gas-permeable foil (BreatheEasy, Diversified Biotech). The cultures were grown for 1000 min (1250 min for Cbi) in a BioTek Power Wave 340 plate reader at 37 °C, shaking. The OD_600_ was measured every 5 min at 600 nm. All experiments were conducted as technical triplicates from biological triplicate. The displayed growth curves are the averages of all nine curves.

### Western blotting

Cells grown in LB-medium were broken in 50 mM K-P_i_ pH 7.5 supplemented with 10% glycerol, 1 mM MgSO_4_, 1 mM phenylmethylsulfonyl fluoride (PMSF) and DNaseI with glass beads in a tissue lyser at 50 hertz. The lysate was centrifuged for 10 min at 20,000×*g* and 4 °C and the supernatant was used for further analysis. The samples analysed by SDS-polyacrylamide gel electrophoresis followed by semi-dry western blotting. The primary antibody was mouse anti-Tetra·His Antibody, BSA-free from Qiagen (Cat.No. 34670) and the secondary antibody was anti-mouse IgG (whole molecule)-alkaline phosphatase conjugate antibody from Sigma-Aldrich (Cat.No. A1902-1ML). The dilutions were 1:2000 and 1:10,000, respectively. The full-length blot from Fig. 1 is included (Supplementary Figure 2h)

### Overexpression and crude membrane vesicle preparation

All BtuM_Td_ variants were overexpressed in *E. coli* MC1061. Overnight pre-cultures in LB-medium supplemented with 100 μg ml^−1^ ampicillin were diluted in a 1:100 ratio and allowed to grow at 37 °C to an OD_600_ of 0.6–0.8. Expression was induced by addition of 0.05% l-arabinose for 3 h. Cells were harvested, washed with 50 mM K-P_i_ pH 7.5, and broken with a Constant Systems cell disruptor at 25 kpsi in 50 mM K-P_i_ pH 7.5 supplemented with 200 μM PMSF, 1 mM MgSO_4_ and DNaseI. Cell debris were removed by centrifugation for 30 min with 25,805×*g* and 4 °C. The supernatant was centrifuged for 2.5 h at 158,420×*g* (average) and 4 °C to collect crude membrane vesicles (CMVs). The CMV pellet was homogenized in 50 mM K-P_i_ pH 7.5 and used for purification.

### Purification of BtuM_Td_ for crystallisation

His-tagged BtuM for crystallisation was solubilised in buffer A (50 mM HEPES/NaOH pH 8, 300 mM NaCl, 0.05 mM cyano-Cbl, 1% *n*-dodecyl-β-maltoside (DDM) and 15 mM imidazole/HCl pH 8.5) for 45 min at 4 °C with gentle movement. Unsolubilized material was removed by centrifugation for 35 min at 219,373×*g* (average) and 4 °C. The supernatant was decanted into a poly-prep column (BioRad) containing 0.5 ml bed volume superflow Ni^2+^-NTA sepharose (GE healthcare) equilibrated with 20 column volumes (CV) buffer A containing additionally 3 mM dithiotreitol (DTT) and incubated for 1 h at 4 °C with gentle movement. Unbound protein was allowed to flow through and the column was washed twice with ten CV buffer A supplemented with 3 mM DTT and 0.35% *n*-nonyl-β-d-glucopyranoside (NG) and 60 mM or 90 mM imidazole/HCl pH 8.5. Bound protein was eluted from the column in four fractions of 0.5 ml (first)—0.7 ml (others) with buffer A supplemented with 3 mM DTT 0.35% NG and 350 mM imidazole/HCl pH 8.5. The sample was centrifuged for 5 min at 20,000×*g* and 4 °C to remove aggregates, and then loaded on a SD200 10/300 Increase SEC column (GE healthcare), which was equilibrated with 30 ml buffer B (50 mM HEPES/NaOH pH 8, 100 mM NaCl, 0.005 mM cyano-Cbl and 0.35% NG) and eluted in the same buffer while monitoring absorption at 280 and 361 nm.

### Purification of His-tagged BtuM_Td_

Purification of His-tagged protein for biochemical analyses was essentially performed as described above with the following adaptations. HEPES was replaced with 50 mM K-P_i_ pH 7 or 7.5 (for ITC and spectral analyses, respectively), NG was replaced with 0.04% DDM, and 100 mM NaCl was used throughout. For purification of the *apo* protein, substrate was omitted from all buffers. For spectral analyses of substrate-bound proteins, substrate was omitted from buffer B.

### Purification of EPEA-tagged BtuM_Td_

EPEA-tagged protein was purified as described above with the following adaptations. CaptureSelect^TM^ C-tagXL Affnity Matrix (Thermo Fisher Scientific) was used. DTT and imidazole were omitted in all steps and 50 mM Tris/HCl pH 7.5 was used instead of K-P_i_. The column was washed once with 10 CV buffer supplemented with 500 mM MgCl_2_. Elution was done in four fractions of 0.5 (first) ml—0.8 ml (others) in buffer containing 2 M MgCl_2_.

### Crystallisation and phasing and structure determination

BtuM_Td_ purified for crystallisation was concentrated to between 1.1 and 1.6 mg ml^−1^ with a 10,000 kDa cut-off Vivaspin concentrator (Sartorius) at 4000×*g* at 2 °C. The initial screening was done using a Mosquito robot (TTP Labtech), and a hit was found after 1 month in the H1 condition (50 mM Tris pH 8.5, 28% (v/v) PEG400) of the MemGold2 screen (Molecular Dimensions) at 4 °C. Larger and better diffracting pyramid-shaped crystals were obtained at 8 °C after 3 to 4 weeks in a crystallization buffer containing 25 mM Tris pH 8.5 and 25 to 30% (v/v) PEG400, 50 mM Tris pH 8.5 and 27 to 30% (v/v) PEG400 or 75 mM Tris pH 8.5 and 29 to 30% (v/v) PEG400, using the sitting drop vapour diffusion method (in MRC Maxi 48-well plate) and a 1:1 mixing ratio (2 μl final drop volume) of mother liquor and protein solution. Phases were obtained from crystals that were soaked for 1 min with 100 mM Tb-Xo4^37^ (Molecular Dimensions) mother liquor solution (0.5 μl added directly to the drop). Diffraction data of the native crystals were collected at the Swiss Light Source (SLS) at PXI (X06SA) beamline (*λ* = 1.000 Å, *T* = 100 K) and two anomalous diffraction datasets were collected at the European Synchrotron Radiation Facility (ESRF) at beamline ID23-1 (*λ* = 1.400 Å and 1.476 Å, *T* = 100 K). Data were processed with XDS^38^ and the two datasets containing anomalous information were merged and subsequently used to solve the structure with ShelX^39^. Autobuild^40^ was used to obtain a starting model, which was refined further with Phenix refine^41^ with manual adjustments done in Coot^42^. The model was used as an input to solve the phase problem for the native dataset, which was carried out with Phaser-MR^43^. The model of the native data was refined iteratively with Phenix refine^41^ and manual adjustments were done in Coot^42^. The Ramachandran statistics for the final model are 99.47% for favoured regions, 0.53% for allowed regions and 0.00% for outliers. A stereo view of 2*F*_o_ – *F*_c_ electron density of the entire structure including the backbone trace molecule, the binding pocket and the Cbl-ligand is provided in Supplementary Figure 5a–c, respectively. All structural figures were prepared with an open-source version of pymol (https://sourceforge.net/projects/pymol/).

### UV–Vis assay to determine decyanation of vitamin B12

All measurements were carried out in a Cary100Bio spectrophotometer (Varian) at room temperature and baseline corrected for buffer B in a quartz cuvette. To monitor the binding of dicyano-Cbi or cyano-Cbl by BtuM_Td_ over time, every minute a spectrum was recorded between 260 and 640 nm for 40 min (Cbi, *n* = 3) or every 20 min for 12 h (Cbl, *n* = 1) at room temperature. For this measurement, a molar protein to substrate ratio of 5:1 (Cbi) or 1:1 (Cbl) was used. To obtain the apparent time constant, *τ*, the absorbance ratio of 369/330 nm was plotted against the time and fitted with a single exponential decay function in Origin 8. Decyanation assays with Cbi were conducted as technical triplicates and errors are standard deviations of the averaged ratios (if not specified otherwise).

### ITC measurement with Cbi

Binding of dicyano-Cbi to purified BtuM_Td_ was measured on a microcal200 ITC (GE healthcare) in high feedback mode. The cell temperature was set to 25 °C with a reference power of 9.5 μcal s^−1^. During the measurement, the sample was stirred at 750 rpm and a 15-fold excess (WT) or 32.5-fold excess (C80S) of Cbi in the syringe was used over the protein concentration in the cell. The data was analysed in Origin and experiments were done as technical triplicates (*n* = 3). The obtained dissociation constants were averaged and the error is the standard deviation of the replicates.

### Mass spectrometry

BtuM_Td_ variants and mutant proteins were purified as described above. BtuM_Td_ proteins were diluted in a 1:1 (v/v) ratio with 0.1% formic acid and 5 μl were injected into an Ultimate 3000-UPLC system (Dionex), connected to a Q-Exactive mass spectrometer (Thermo Fisher Scientific) and separated on a 2.1 mm × 50 mm Acquity UPLC BEHC18, 1.7 μm (Waters). Solvent A was H_2_O with 0.1% formic acid and solvent B was acetonitrile with 0.1% formic acid. The following mobile phase gradient was delivered at a flow rate of 0.6 ml min^−1^ starting with a mixture of 60% solvent B for 1 min. Solvent B was increased to 90% over 5 min with a linear gradient and kept at this concentration for 5 min. Solvent B was reduced to 60% in 0.1 min and kept for 3.9 min resulting in a total elution time of 15 min. The column temperature was kept constant at 40 °C. The mass spectrometer was operated in positive mode. Full scan MS spectra were acquired for 10 min from *m*/*z* 1000 to 2000 at a target value of 1 × 10^6^ and a max IT of 500 ms with a resolution of 140,000 at *m*/*z* 200. Scans were averaged using Xcalibur 4.0.27.42 Qualbrowser and the isotopically resolved MS spectrum was de-convoluted using the built-in Xtract algorithm.

### Data availability

Data supporting the findings of this manuscript are available from the corresponding author upon reasonable request. Atomic coordinates and structure factors for the crystal structure of BtuM_Td_ have been deposited in the Protein Data Bank under the accession code 6FFV. The mass spectrometry data have been deposited to the ProteomeXchange Consortium via the PRIDE partner repository with the dataset identifier PXD010024.

## Electronic supplementary material

Supplementary Information

Description of Additional Supplementary Files

Supplementary Data 1
