# Supplementary material for: Cysteine-mediated decyanation of vitamin B12 by the predicted membrane transporter BtuM
Source: Nat Commun. 2018 Aug 2;9:3038. doi: 10.1038/s41467-018-05441-9 (PMC6072759; doi:10.1038/s41467-018-05441-9)
Supplement: Supplementary file 1 — Supplementary Information [file 41467_2018_5441_MOESM1_ESM.pdf]

Cysteine-mediated decyanation of vitamin B12 by the predicted membrane transporter BtuM

Rempel S., et al.

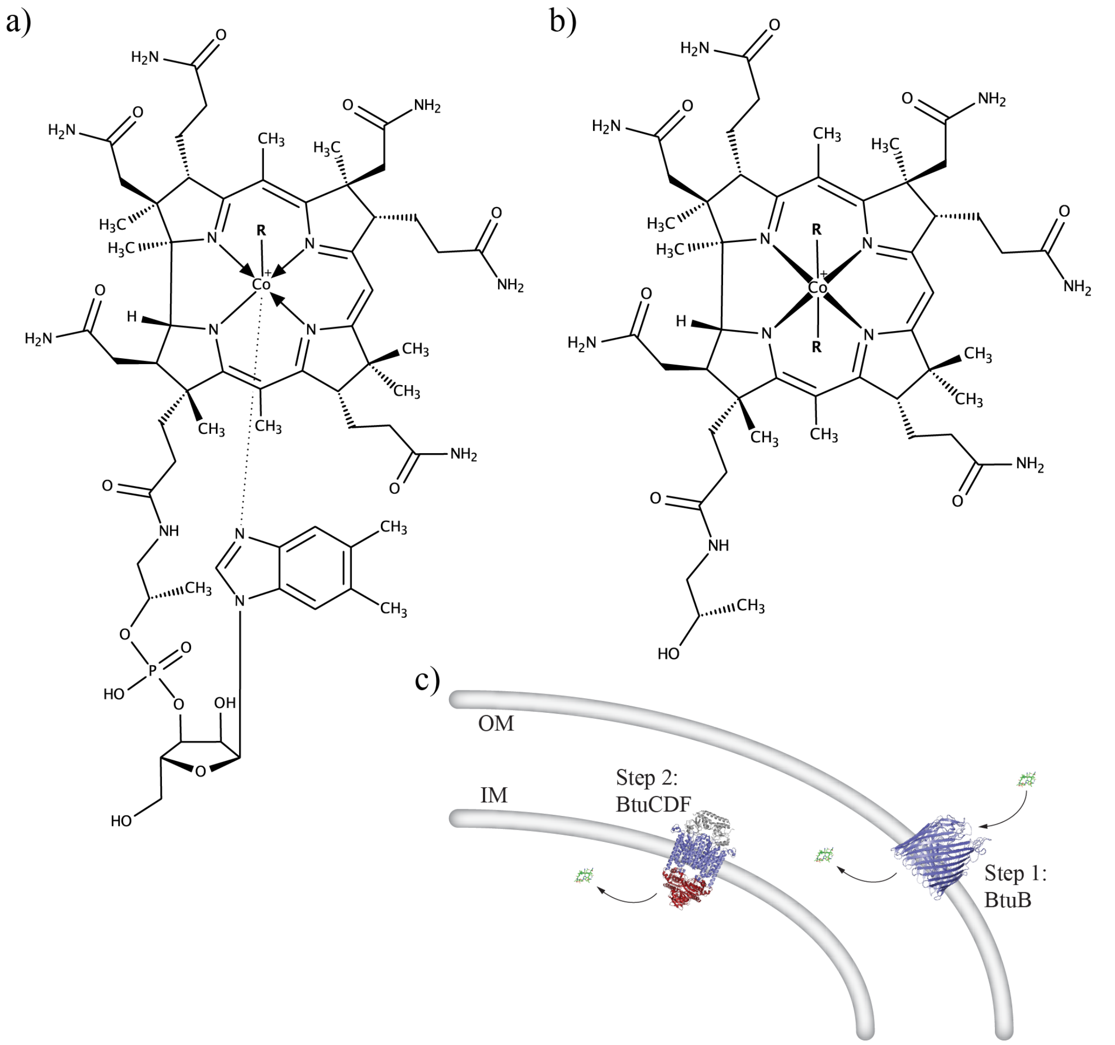

Supplementary Figure 1 Structure of cobalamin and cobinamide

(a) Structural formula of cobalamin (Cbl) where R can be a cyano group (cyano-Cbl), a hydroxyl group (OH-Cbl), a methyl-group (CH3-Cbl) or a 5'-deoxyadenosyl group (ado-Cbl). The latter two are two of three biologically active variants of the vitamin. The third variant is found in epoxyqueuosine oxidoreductases, which bind Cbl in an 'open conformation' where R is a water and Cbl is bound in the base-off conformation and its cobalt ion is kept penta-coordinate (in contrast to the 'normal' hexa-coordinate) and thus in its divalent state. **(b)** The Cbl precursor cobinamide (Cbi) has two variable groups, R. In this study, di-cyano Cbl was used. **(c)** The uptake of Cobalamin in *E. coli* requires the translocation of Cbl (green) over the outer (OM) and inner (IM) membrane. BtuB is the TonB-dependent outer membrane active transporter (PDB: 2GSK) and BtuCDF (PDB: 4FI3) is a type II ABC-importer. Together they form the full BtuBCDF transport pathway. In our deletion strain, *E. coli* ΔFEC, the *btuB* gene locus is still present.

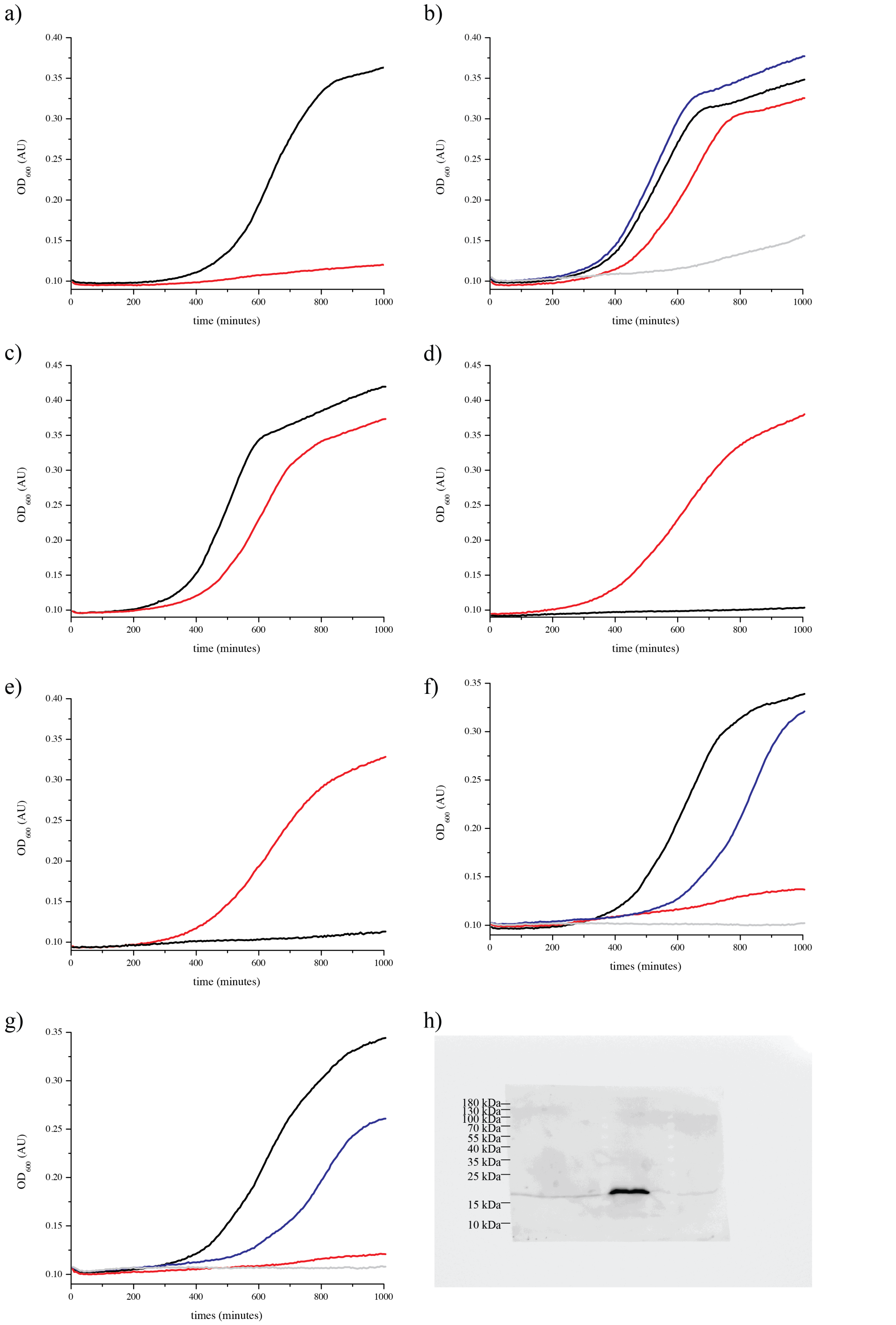

Supplementary Figure 2 Growth assay in the presence of OH-Cbl and Cbi and using extreme Cbl concentrations

All growth curves are averages of biological triplicates each consisting of technical triplicates. **(a)** *E. coli* ΔFEC without expression plasmid can grow in the presence of 50 μg/ml L-methionine (black line)

but not in the presence of 1 nM Cbl (red line). **(b)** Growth of *E. coli* ΔFEC expressing EPEA-tagged versions of $BtuM_{Td}$. In the presence of 50 μg/ml L-methionine both wild-type and C80S mutant grow (black and blue line, respectively), whereas in the presence of 1 nM Cbl only $BtuM_{Td}$ (red line) can grow and the C80S mutant does not exhibit substantial growth (grey line). **(c)** Growth of tag-less $BtuM_{Td}$ expressing cells shows that the His-tag does not affect activity of $BtuM_{Td}$ *in vivo* in the presence of either L-methionine (black line) or Cbl (red line). **(d)** Growth assay in the presence of 0.1 nM OH-Cbl of cells expressing BtuCDF (red line) and control carrying the empty expression vector (black line). **(e)** Growth assay in the presence of 0.1 nM OH-Cbl of wild-type $BtuM_{Td}$ expressing cells (red line) and the C80S mutant (black line). **(f)** At Cbl concentrations of 5 nM both BtuCDF expressing cells (black line) and empty expression vector carrying cells (blue line) grow whereas 0.01 nM Cbl is insufficient to sustain grow for either BtuCDF expressing cells (red line) or cells carrying pBAD24 (grey line). **(g)** For cells expressing His-tagged $BtuM_{Td}$ (black and red lines) and $BtuM_{Td}$_C80S (blue and grey lines) under the same conditions as in e) we observe the same behaviour. **(h)** Full length western blot of the inset in **Figure 1 b**.

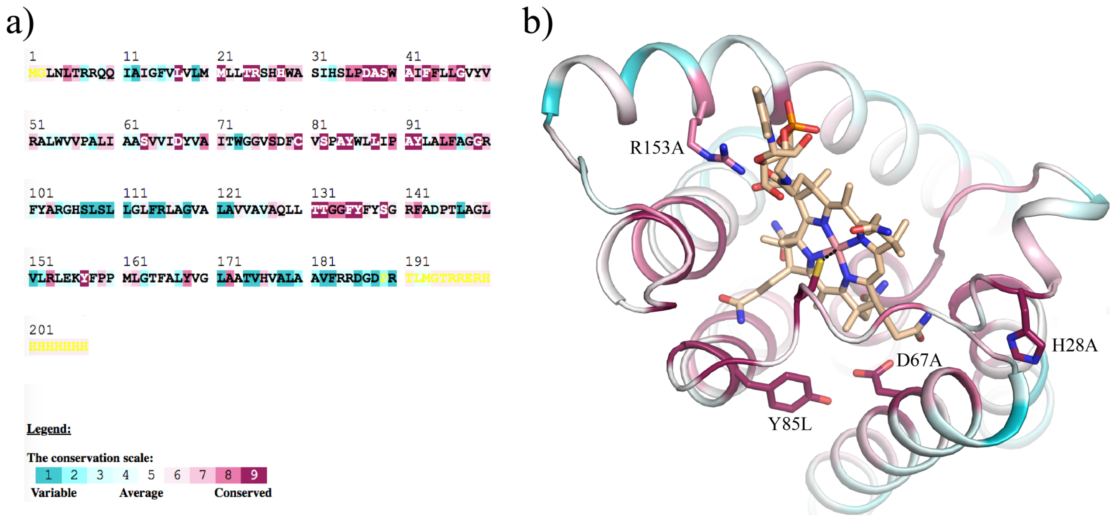

Supplementary Figure 3 Sequence conservation in the BtuM family

(a) Amino acid sequence of $BtuM_{Td}$ with colour key showing the degree of conservation (yellow residues with insufficient data). Residue Cys80 is completely conserved. **(b)** View from the periplasmic side of the membrane on the $BtuM_{Td}$ binding pocket. The colouring of residues by conservation was mapped on the structure using the ConSurf Server. Four residues were chosen and mutant protein variants were constructed, $BtuM_{Td}$_H28A, D67A, Y85L, and R153A.

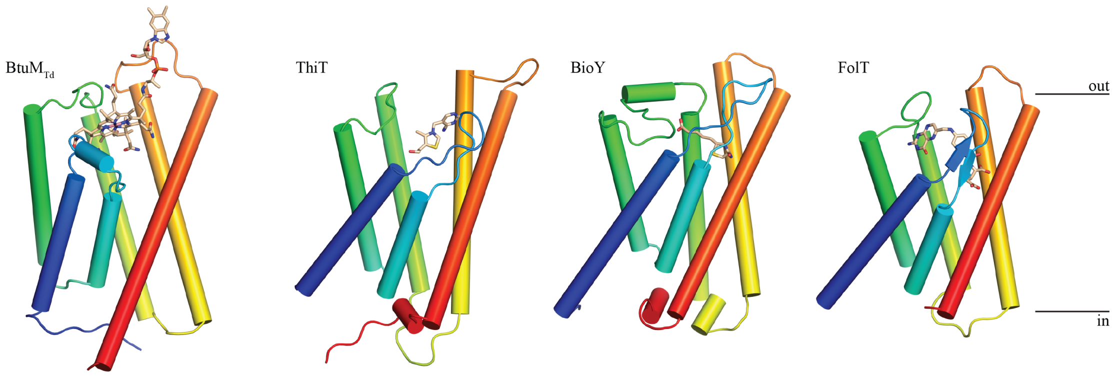

Supplementary Figure 4 Comparison of BtuM$_{Td}$ to three other S-components

Structural alignment of BtuM$_{Td}$ with three S-components ThiT, BioY and FolT (pdb-codes 3RLB, 4DVE and 5D0Y, respectively) shows that the overall fold is the same. Also, the substrate-binding site is located at the same position. RMSD values between are listed in **Supplementary Table 1**.

a)

b)

c)

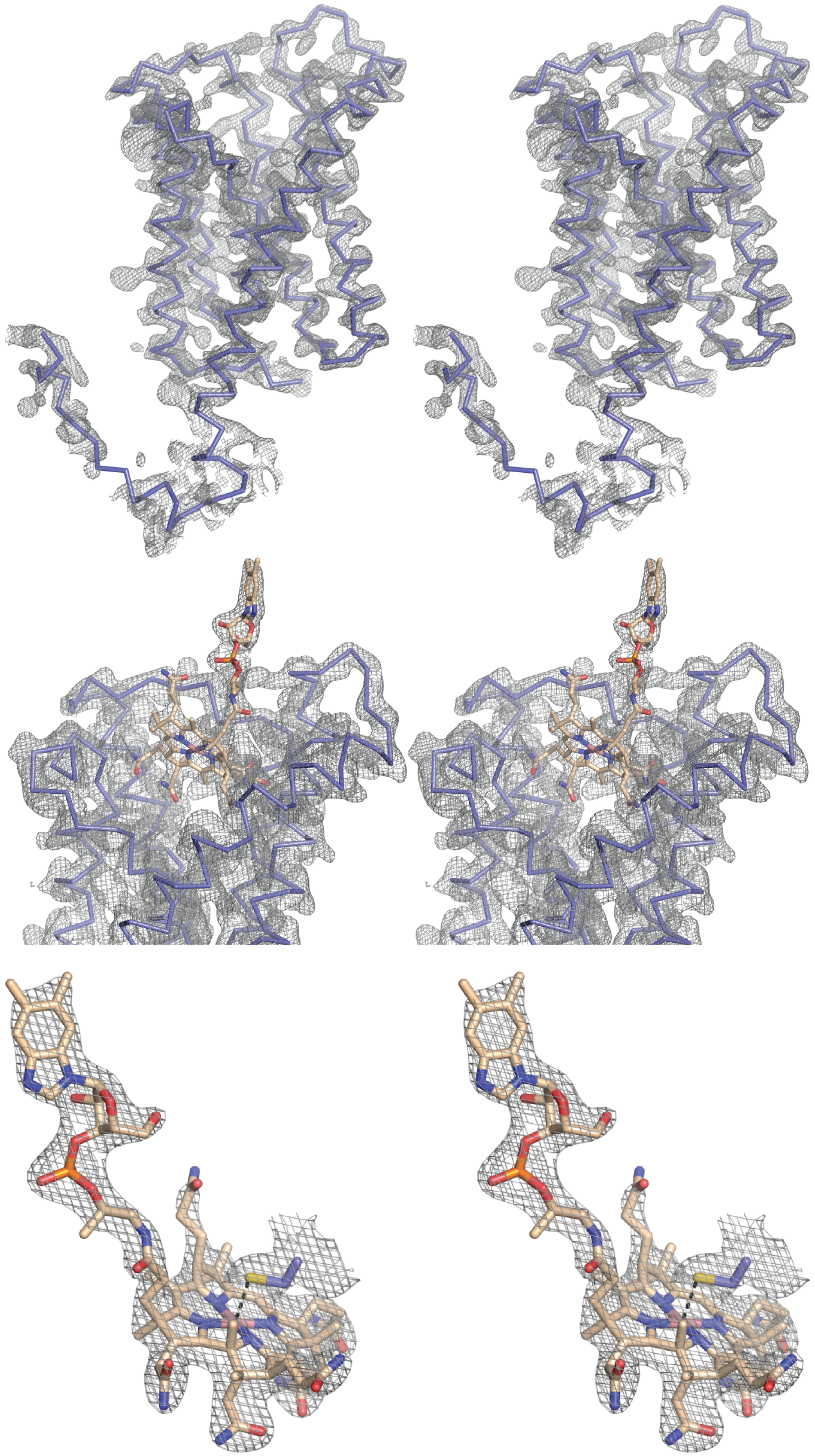

Supplementary Figure 5 Stereo view images of the main chain traced electron density of $BtuM_{Td}$ and its binding pocket and vitamin B12

(a) Stereo view image of the full-length ribbon traced model with its corresponding 2fo-fc density map (grey) at 2σ (residues 3-182 and 202-207) and 0.5σ (residues 1-2 and 183-201). **(b)** The same model as in (a) focused on the binding pocket including Cbl and its corresponding 2fo-fc density (grey) at 2σ. **(c)** Stereo view on Cbl and Cys80 with their 2fo-fc density map (grey) at 2σ.

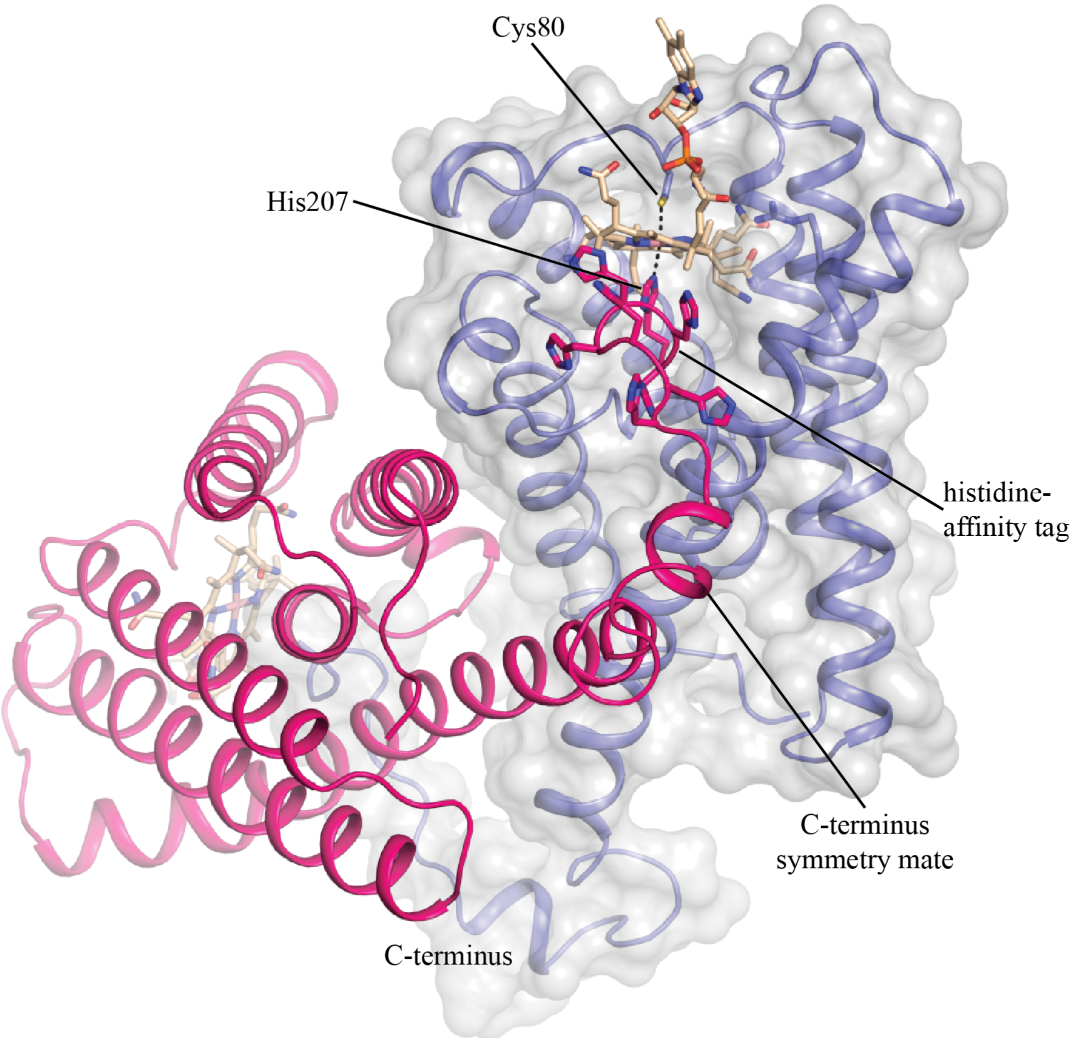

Supplementary Figure 6 BtuM$_{Td}$ and its neighbouring symmetry mate in the crystal

The two symmetry mates (blue and pink) align almost antiparallel allowing the C-terminal histidine-affinity tags to mutually enter into each other's binding pocket. The last His207 then binds the Co-ion of Cbl.

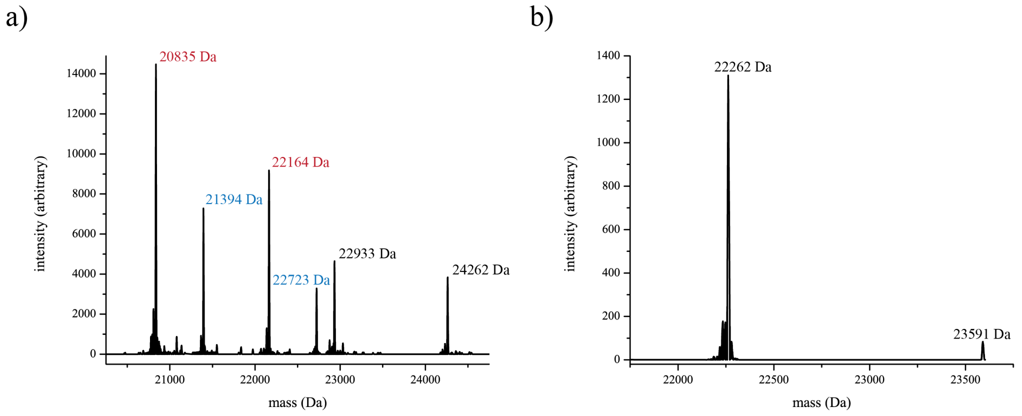

Supplementary Figure 7 Mass spectrometry analysis of BtuM$_{Td}$_cHis8 and BtuM$_{Td}$_cEPEA bound to co-purified Cbl showing the loss of the β-ligand

(a) BtuM$_{Td}$_cHis (native mass of 22905 Da) bound to Cbl yields multiple peaks. These peaks can be separated into three pairs of which the higher mass corresponds to the substrate-bound protein and the lower mass to the *apo* form. The mass differences are 1329 Da, which corresponds to the mass of Cbl without β-ligand. The masses labelled in black (22933 Da and 24262 Da) are the native protein with a formylated first methionine (adding 28 Da). The pair labelled in red (20835 Da and 22164 Da) and the pair labelled in blue (21394 Da and 22723 Da) correspond to truncated forms with loss of a 2098 Da C-terminal peptide (red) and 1539 Da C-terminal peptide (blue). The amino acid sequences of the lost peptides of the truncated versions are LMGTRRERHHHHHHHH (red) and RERHHHHHHHH (blue). **(b)** BtuM$_{Td}$_cEPEA (native mass 22234 Da) also shows the loss of the β-ligand . We observe the mass for formylated *apo* protein (22262 Da) and formylated substrate-bound protein (23591 Da). The difference between the two (1329 Da) is the mass of decyanated Cbl .

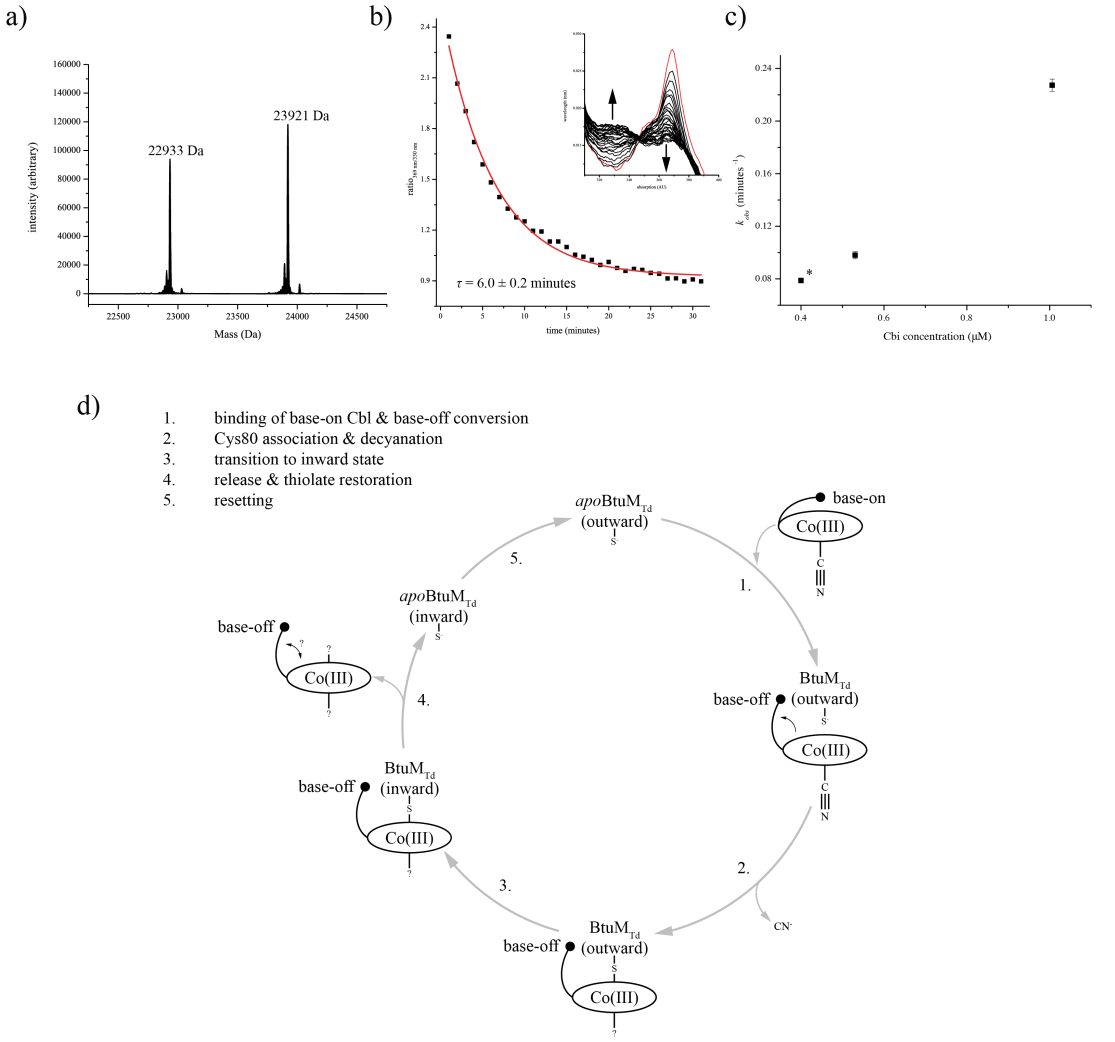

Supplementary Figure 8 High resolution mass spectrum of BtuM$_{Td}$ bound to Cbi and decyanation of Cbi by His-tagged BtuM$_{Td}$ and proposed mechanism for decyanation

(a) The formylated *apo* protein with M$_w$ of 22933 Da (similar to **Supplementary Figure 7 a**) and a peak for the substrate bound protein (23921 Da) are visible. The difference of the two masses is 988 Da, close to the mass of decyanated Cbi of 990 Da; because dicyano-Cbi was added during the purification (Mw of 1042 Da) the data indicates that the protein removed both cyano groups. **(b)** Spectral changes of a 5:1 His-tagged BtuM$_{Td}$ (0.5 – 1.3 µM) to Cbi molar ratio mixture were monitored over time (starting spectrum red line). The absorption increased at 330 nm and decreased at 369 nm. These changes are consistent with removal of cyanide from the substrate. His-tagged BtuM$_{Td}$ and EPEA-tagged BtuM$_{Td}$ behaved similarly (compare **Figure 3 b and c**) **(c)** Decyanation assay with a fixed concentration (4 µM) of EPEA-tagged BtuM$_{Td}$ with increasing concentrations of Cbi (* value from single experiment, other points averaged from triplicates with standard deviation as the error of the mean) showing that the decaynation process follows the kinetics of a pseudo-first order binding reaction. **(d)** Possible mechanism of decyanation. Decyanation of the substrate that is in its base-on conformation at physiological pH in the periplasm with a trivalent Co-ion, binds to BtuM$_{Td}$ with its binding pocket exposed to the extracellular side (step 1). The cysteine replaces the α-ligand. We assume that the side chain of Cys80 is in its thiolate form allowing it to donate one electron to allow for

a simple one-step reductive decyanation (step 2). We propose that the Co ion remains in its trivalent state (hexa-coordinate) throughout the reaction. We hypothesize that release of Cbl on the intracellular side (step 3) of the membrane and breaking of the Co-S bond is achieved by the reducing environment of the cell allowing the Cys80 to return in its thiolate state (step 4) making it accessible to undergo another reaction once the membrane protein has transitioned back to the outward facing conformation (step 5).

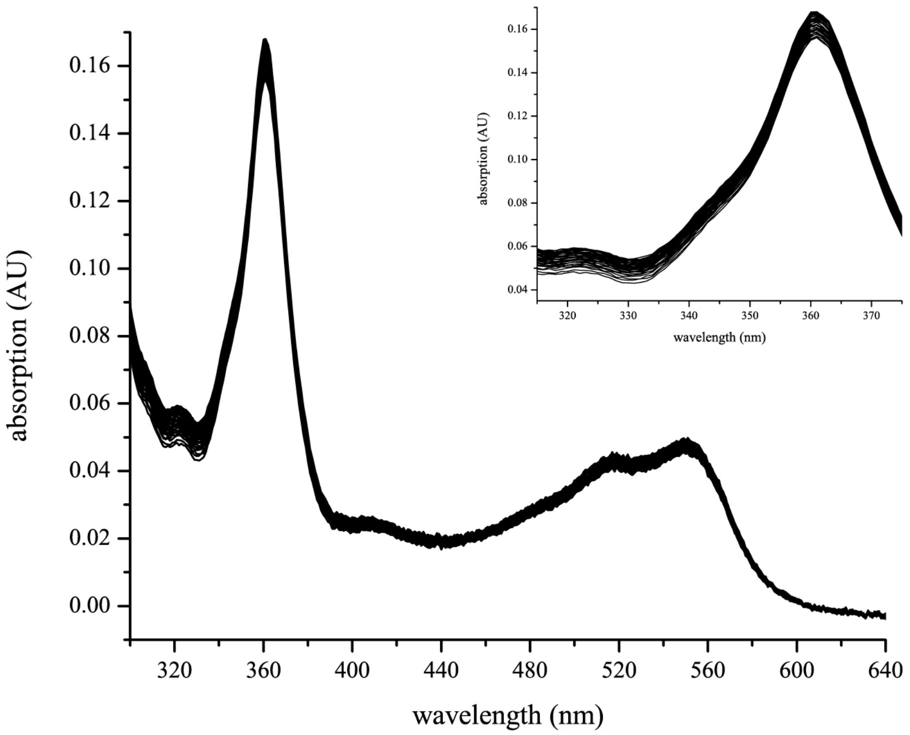

Supplementary Figure 9 Monitoring cyano-Cbl binding by $\mathrm{BtuM_{Td}}$ by spectral changes

Similar to the experiment in **Supplementary Figure 8 b** spectral changes in $\mathrm{BtuM_{Td}}$_cHis8 upon substrate binding were followed over time. A molar ratio of substrate to protein of 1:1 was used and spectra were taken every 20 minutes for 12 hours. Because binding would lead to the spectral changes observed in **Figure 2 b**, we conclude that we do not observe binding.

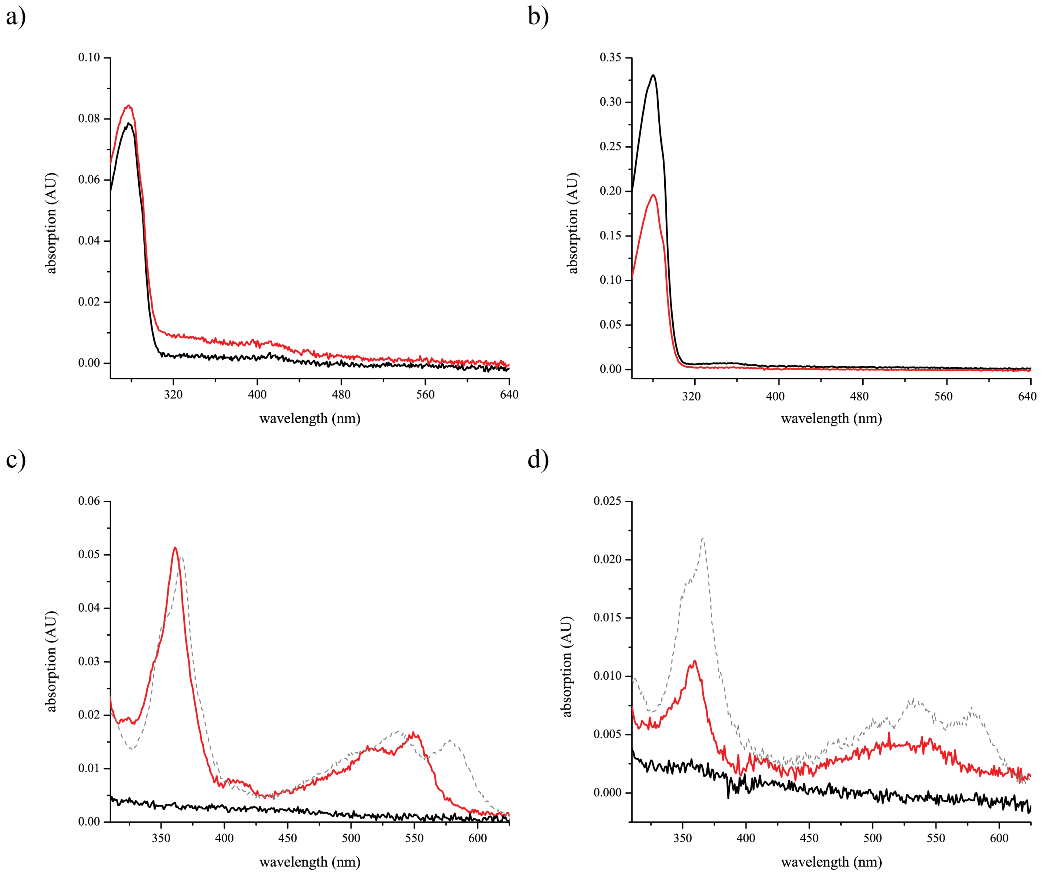

Supplementary Figure 10 Absorption spectra of *apo* BtuM$_{Td}$ and mutants C80A and C80S

(a) Absorption spectra of *apo* EPEA-tagged BtuM$_{Td}$ (black line) and BtuM$_{Td}$_C80S (red line) showing that the protein purifies in its *apo* state when no substrate is added. **(b)** The absorption spectra of His-tagged BtuM$_{Td}$_C80A (black line) and C80S (red line) purified in the presence of Cbl show that no substrate is bound. **(c)** and **(d)** The two mutant variants BtuM$_{Td}$_C80A and C80S were purified in conditions where wild-type BtuM$_{Td}$ binds Cbl and Cbi. BtuM$_{Td}$_C80A **(c)** and BtuM$_{Td}$_C80S **(d)** did not bind Cbl (black line, same data as in b) but still bound Cbi (red line). Binding of Cbi did not lead to the spectral changes as observed for the native protein (compare **Figure 2 b and c**). Spectra of 2 μM (c) and 1 μM (d) unbound Cbi (dashed grey line) are included for comparison.

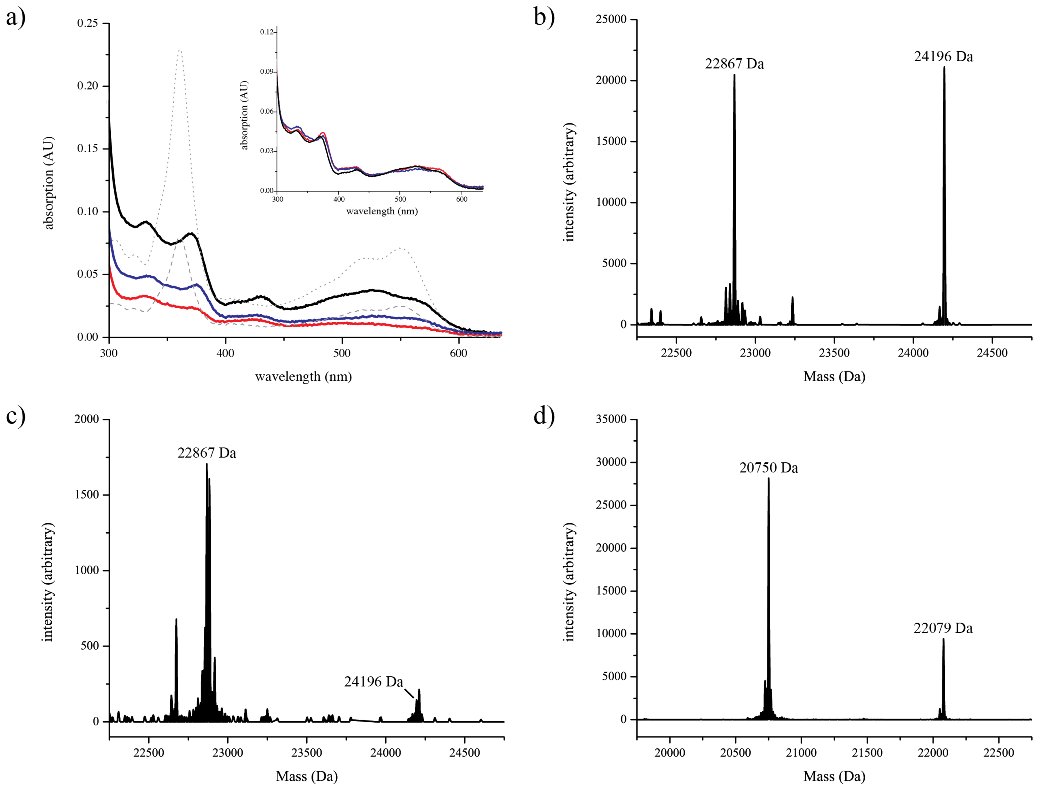

Supplementary Figure 11 Absorption spectra and high-resolution mass spectra of BtuM$_{Td}$ mutants bound to Cbl

(a) The absorption spectra of Cbl bound to BtuM$_{Td}$_H28A (black line, 8.7 µM), Y85L (red line, 3.1 µM), and R153A (blue line, 4.4 µM) show the characteristic changes of cysteine binding and decyanation. For comparison spectra of free Cbl are shown at concentrations of 3 µM and 8.5 µM, grey dashed and dotted line, respectively. The inset shows scaled spectra of the mutants to emphasize that spectral changes caused by binding to mutants of BtuM$_{Td}$ are essentially the same (inset) and apparent differences are caused by different concentrations. **(b)** Mass spectrum of BtuM$_{Td}$_H28A shows two peaks corresponding to the formylated *apo* protein (22867 Da) and the formylated, Cbl-bound protein (24196 Da). The mass difference of 1329 Da is consistent with decyanation. **(c)** Same for the Y85L mutant **(d)** Same for the R153A mutant.

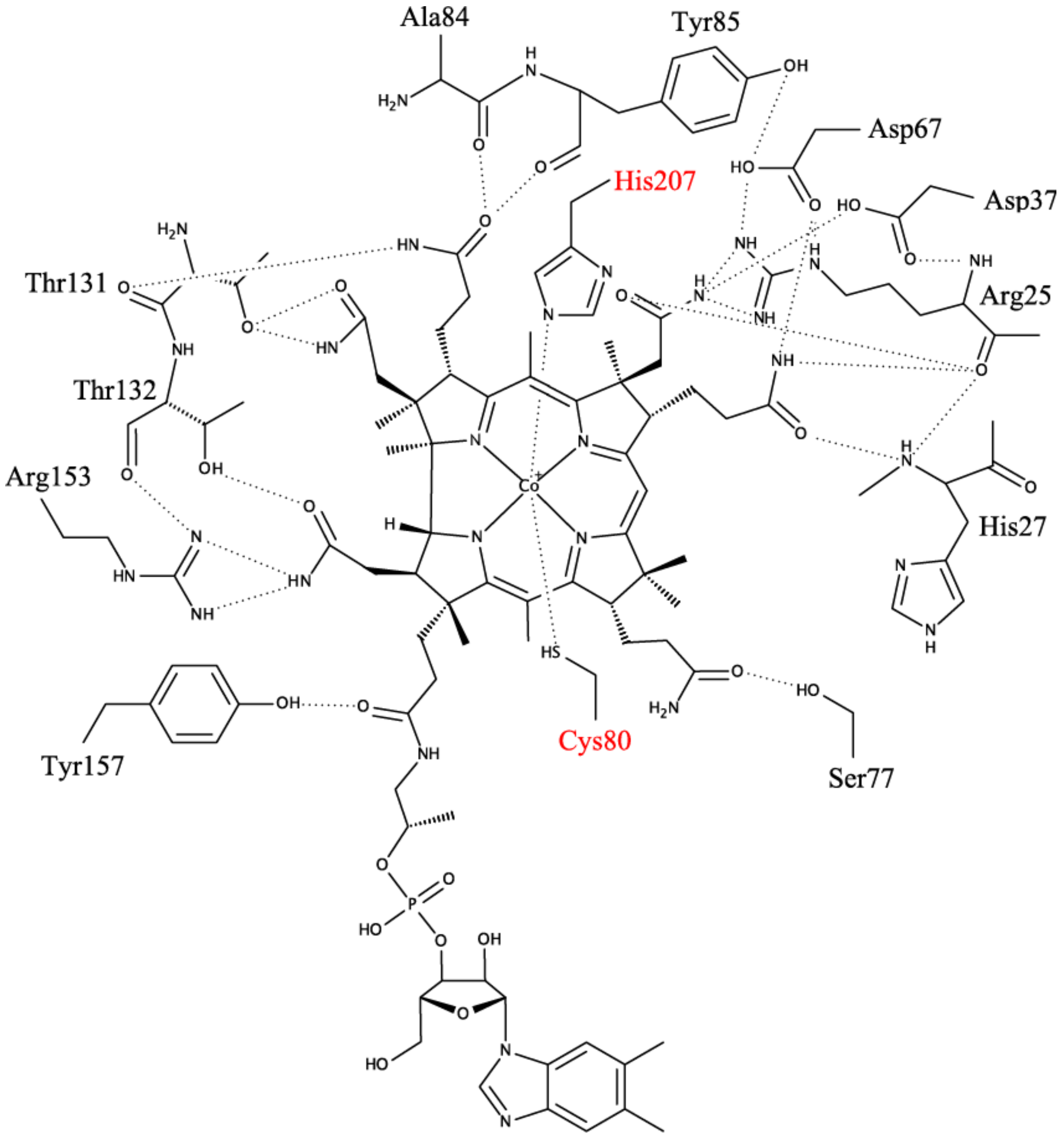

Supplementary Figure 12 Depiction of hydrogen bond network in the binding pocket of Cbl with $\mathbf{BtuM_{Td}}$

Next to the major interactions of the sulphur and nitrogen of Cys80 and His207 with the cobalt ion of Cbl there are a variety of side chain and backbone interactions with corrin-ring decorating moieties.

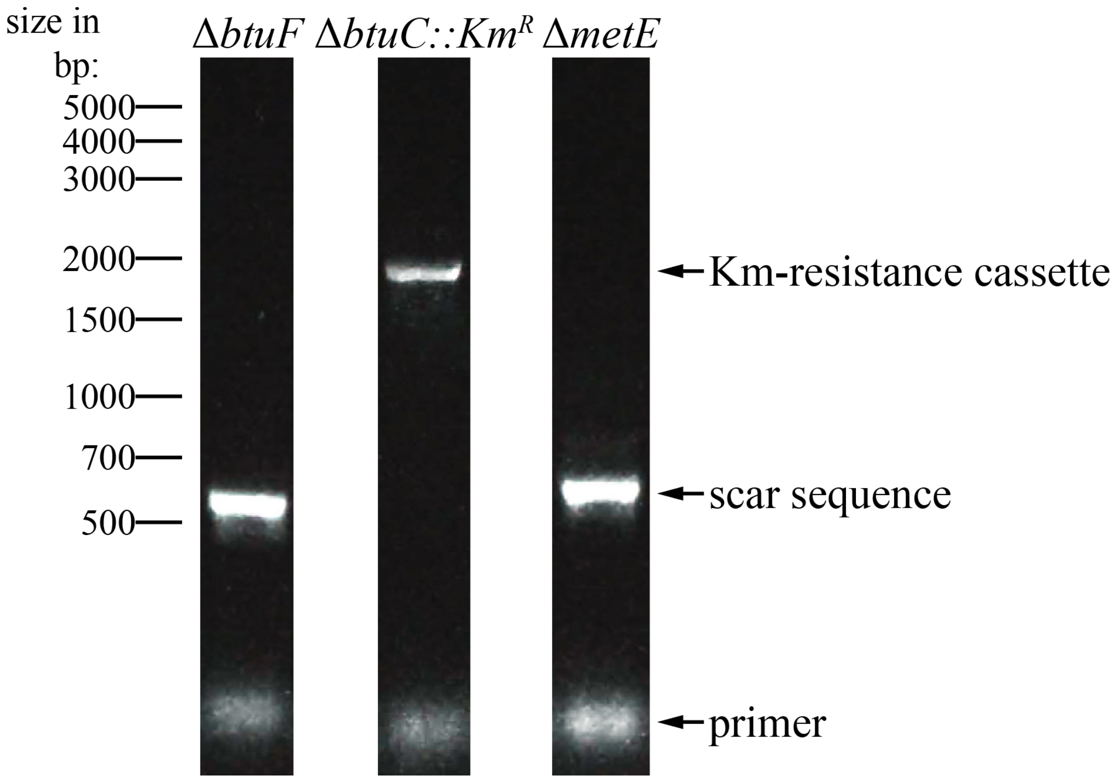

Supplementary Figure 13 Colony PCR of *E. coli* ΔFEC displaying correct deletion of target genes

An agarose gel of a colony PCR using primers designed in such way (see Materials and Methods) that wild-type loci can be distinguished from either the scar sequence left after FLP-treatment or the kanamycin resistance cassette after P1 insertion. The expected PCR product sizes for the native loci are 981 base pairs (bp) for *btuC*, 801 bp for *btuF* and 2262 bp for *metE*.

Supplementary Table 1 RMSD value matrix (in Å) of the structural comparison between full length $BtuM_{Td}$ and ThiT and BioY and FolT

	**$BtuM_{Td}$**	**ThiT**	**BioY**	**FolT**
**$BtuM_{Td}$**	0.0	3.1	3.2	3.1
**ThiT**	3.1	0.0	2.8	2.6
**BioY**	3.2	2.8	0.0	2.9
**FolT**	3.1	2.6	2.9	0.0

Supplementary Table 2: Primer list used in this study.

**Primer name**	**Sequence (5’-3’)**
BtuM_opt_NcoI_frwd	GGTCCATGGGTCTGAATC
BtuM_NcoI_long_frwd	GGTCCATGGGTCTGAATCTGACCCGTCGTCAGCAGATTGC
BtuM_Td_opt_C80S_frwd	GTTAGCGATTTTTCTGTTAGTCCGGC
BtuM_Td_opt_C80S_rev	GCCGGACTAACAGAAAAATCGCTAAC
BtuM_Td_opt_C80A_frwd	GGTGTTAGCGATTTTGCGGTTAGTCCGGCATATTG
BtuM_Td_opt_C80A_rev	CAATATGCCGGACTAACCGCAAAATCGCTAACACC
BtuM_Td_opt_H28A_frwd	GACCCGTAGCCATGCTTGGGCAAGCATTC
BtuM_Td_opt_H28A_rev	GAATGCTTGCCCAGCGATGGCTACGGGTC
BtuM_Td_opt_D67A_frwd	GATTGCAGCAAGCGTTGTTATTGCTTATGTTG
BtuM_Td_opt_D67A_rev	CAGGTAATTGCAACATAAGCAATAACAACG
BtuM_Td_opt_Y85L_frwd	GTTAGTCCGGCACTTTGGCTGCTG
BtuM_Td_opt_Y85L_rev	GCAGCCAAAGTGCCGGACTAACAC
BtuM_Td_opt_R153A_frwd	CAGGTCTGGTGCTGGCTCTGGAAAAATAC
BtuM_Td_opt_R153A_rev	GTATTTTTCCAGAGCCAGCACCAGACCTG
BtuM_Td_opt_no-cHis8_rev	GCCAAGCTTTCATTAACGTTCACGACGGG
BtuM_Td_cEPEA-HindIII_rev	GATAAGCTTTCATTATGCCTCTGGTTCACGTTCACGACGGG
BtuC_frwd	GCAGGAGGAATTCACCATGCTGACACTTGCCCGC
BtuC_rev	GAATTCCTCCTATTGATTACTAACGTCCTGCTTTTAACAATAACCAG
BtuF_frwd	GACGTTAGTAATCAATAGGAGGAATTCACCATGGCTAAGTCACTGTTCAGG
BtuF_rev	GCCAAAACAGCCAAGCTTTTACTAATCTACCTGTG

	AAAGCGCATTAC
pBAD24_frwd	TTAAAGCTTGGCTGTTTTGGCG
pBAD24_rev	GGTGAATTCCTCCTGCTAGC
Seq_frwd	CTCTACTGTTTCTCCATACCCG
Seq_rev	GCTGAAAATCTTCTCTCATCCG
