# Supplementary material for: Cysteine-mediated decyanation of vitamin B12 by the predicted membrane transporter BtuM
Source: Nat Commun. 2018 Aug 2;9:3038. doi: 10.1038/s41467-018-05441-9 (PMC6072759; doi:10.1038/s41467-018-05441-9)
Supplement: Supplementary file 2 — Description of Additional Supplementary Files [file 41467_2018_5441_MOESM2_ESM.docx]

**Description of Additional Supplementary Files**

File Name: Supplementary Data 1

Description: Organisms with genes encoding BtuM homologs. The only organism encoding an ECF-module is marked (*).
